# Supplementary material for: RBFOX2 modulates a metastatic signature of alternative splicing in pancreatic cancer
Source: Nature. Author manuscript; Available in PMC 2023 Jun 7. (PMC10156590; doi:10.1038/s41586-023-05820-3)
Supplement: 1897967 Supplementary Info Guide [file NIHMS1897967-supplement-1897967_Supplementary_Info_Guide.docx]

**Supplementary Information Guide**

- **Supplementary Figures 1-18:** Immunoblot, Gel Scans, and Lung Histology raw data
- **Supplementary Table 1:** Genetic alterations and clinical data of PDA patients
- **Supplementary Table 2:** Differentially spliced events in PDA patient samples, primary tumors versus metastatic tumors
- **Supplementary Table 3:** Sequence motif enrichment analysis
- **Supplementary Table 4:** Reactome analysis
- **Supplementary Table 5:** Differentially gene expression changes in PDA patient samples, primary tumors versus metastatic tumors
- **Supplementary Table 6:** Differentially spliced events in RBFOX2 manipulated cell lines
- **Supplementary Table 7:** Comparisons of RBFOX2 target genes to known RBFOX2 target genes
- **Supplementary Table 8:** Serine-threonine kinome analysis for MPRIP isoforms
- **Supplementary Table 9:** Mass spectrometry analysis for MPRIP isoforms
- **Supplementary Table 10:** sgRNAs sequences
- **Supplementary Table 11:** PCR primers sequences
- **Supplementary Table 12:** Primary and secondary antibodies.
