# Supplementary material for: RBFOX2 modulates a metastatic signature of alternative splicing in pancreatic cancer
Source: Nature. Author manuscript; Available in PMC 2023 Jun 7. (PMC10156590; doi:10.1038/s41586-023-05820-3)
Supplement: 1897967 RS [file NIHMS1897967-supplement-1897967_RS.pdf]

nature portfolio

Corresponding author(s): Rotem Karni

Last updated by author(s): 11.01.23

Reporting Summary

Nature Portfolio wishes to improve the reproducibility of the work that we publish. This form provides structure for consistency and transparency in reporting. For further information on Nature Portfolio policies, see our Editorial Policies and the Editorial Policy Checklist.

Statistics

For all statistical analyses, confirm that the following items are present in the figure legend, table legend, main text, or Methods section.

n/a	Confirmed	
☐	☒	The exact sample size (n) for each experimental group/condition, given as a discrete number and unit of measurement
☐	☒	A statement on whether measurements were taken from distinct samples or whether the same sample was measured repeatedly
☐	☒	The statistical test(s) used AND whether they are one- or two-sided<br>*Only common tests should be described solely by name; describe more complex techniques in the Methods section.*
☐	☒	A description of all covariates tested
☐	☒	A description of any assumptions or corrections, such as tests of normality and adjustment for multiple comparisons
☐	☒	A full description of the statistical parameters including central tendency (e.g. means) or other basic estimates (e.g. regression coefficient) AND variation (e.g. standard deviation) or associated estimates of uncertainty (e.g. confidence intervals)
☐	☒	For null hypothesis testing, the test statistic (e.g. F, t, r) with confidence intervals, effect sizes, degrees of freedom and P value noted<br>*Give P values as exact values whenever suitable.*
☒	☐	For Bayesian analysis, information on the choice of priors and Markov chain Monte Carlo settings
☒	☐	For hierarchical and complex designs, identification of the appropriate level for tests and full reporting of outcomes
☒	☐	Estimates of effect sizes (e.g. Cohen's d, Pearson's r), indicating how they were calculated

Our web collection on statistics for biologists contains articles on many of the points above.

Software and code

Policy information about availability of computer code

Data collection	Nikon-TL (fluorescence microscopy images), BIORAD ChemiDoc XRS+ System (immunoblot blots and PCR gels), IncuCyte (wound healing), 2200 TapeStation, Illumina NextSeq 2000 system, LabChip®GX microfluidics platform, Step one plus real-time PCR system-Applied Biosystems, Spinning Disk Confocal Microscope - Nikon Instruments Inc., Aperio Digital Pathology Slide Scanners - Leica Biosystems, real-time cell imaging system - IncuCyte Live cell, Q Exactive Plus mass spectrometer - Thermo Fisher Scientific, nanoflow UHPLC instrument Ultimate 3000 Dionex - Thermo Fisher Scientific.
Data analysis	Bioconductor (v3.7) within the R (v3.5.1) programming environment, GraphPad Prism (version 9.5.0.730) for data analysis and plots, R's prcomp function for PCA analysis, PSI-Sigma (version 1.9c) for splicing analysis, Reactome database for pathway enrichment, XSTREME package (version 5.5) for motif analysis, NIS Elements (version 4.13) imaging software, Analysis software (IncuCyte Cat No 4400 version 2022A) for wound healing analysis, MaxQuant computational platform (version 2.0.3.0) for mass spec analysis, LabChip GX Reviewer software (version 5.3.2115.0) for splicing validations and quantifications, STAR v 2.5.3a. CHOP CHOP for sgRNAs design., AlphaFold for structure analysis. DESeq2 for gene expression analysis.

For manuscripts utilizing custom algorithms or software that are central to the research but not yet described in published literature, software must be made available to editors and reviewers. We strongly encourage code deposition in a community repository (e.g. GitHub). See the Nature Portfolio guidelines for submitting code & software for further information.

Data

Policy information about availability of data

All manuscripts must include a data availability statement. This statement should provide the following information, where applicable:

- Accession codes, unique identifiers, or web links for publicly available datasets
- A description of any restrictions on data availability
- For clinical datasets or third party data, please ensure that the statement adheres to our policy

RNA-seq data generated as part of this study is deposited ito the BioProject under accession number PRJNA797585.
RNA-seq data of PDA patients are available at European Genome Phenome Archive https://www.ebi.ac.uk/ega/home (Study ID EGAS00001002543) databases IDs: EGAD00001003584, EGAD00001004548, EGAD00001005799, EGAD00001006081.
XSTREME database (Ray2013 Homo sapiens), Reactome database (Homo sapiens), Ensembl gene annotation Human (GRCh38.p13, Homo sapiens).

Field-specific reporting

Please select the one below that is the best fit for your research. If you are not sure, read the appropriate sections before making your selection.

☒ Life sciences ☐ Behavioural & social sciences ☐ Ecological, evolutionary & environmental sciences

For a reference copy of the document with all sections, see nature.com/documents/nr-reporting-summary-flat.pdf

Life sciences study design

All studies must disclose on these points even when the disclosure is negative.

Sample size	For experiments in mice, group sizes were determined by power analysis on prior data collected using the same experimental procedures (Tavazoie et.al 2008, Golan, T. et al. 2017) and calibration experiments we performed. Experiments were designed to detect differences greater than 20% at a significance of $p<0.05$. For all mouse experiments (including tumor growth and metastatic model) minimum n=4 mice was used. All the exact sample sizes are stated in the figure legends.
Data exclusions	No data were excluded.
Replication	The experiments were repeated multiple times (2-4), with 2-4 biological replicates each time, and the results were consistent across all the trials. The majority of the assays were conducted three times, with three biological replicates each time, which demonstrates the reproducibility of the data. All attempts at replication in this study were successful.
Randomization	In the in vivo experiments the animals were randomly divided into different groups. For immunofluorescence and immunohistochemistry staining, the fields of view were randomly chosen. For other experiments, where the samples were not randomly assigned, there was no need for group allocation or randomization because all samples were consistently and independently measured in a controlled manner.
Blinding	In the mouse experiments, the experimenters were not blinded to the treatment groups, as they administered the treatments and measured the tumors themselves.<br>In vitro cell culture experiments, blinding is not applicable because the researchers need to verify samples and controls for each experiment. However, whenever feasible, a second researcher confirmed the results.

Reporting for specific materials, systems and methods

We require information from authors about some types of materials, experimental systems and methods used in many studies. Here, indicate whether each material, system or method listed is relevant to your study. If you are not sure if a list item applies to your research, read the appropriate section before selecting a response.

Materials & experimental systems

n/a	Involved in the study	
☐	☒	Antibodies
☐	☒	Eukaryotic cell lines
☒	☐	Palaeontology and archaeology
☐	☒	Animals and other organisms
☒	☐	Human research participants
☒	☐	Clinical data
☒	☐	Dual use research of concern

Methods

n/a	Involved in the study	
☒	☐	ChIP-seq
☒	☐	Flow cytometry
☒	☐	MRI-based neuroimaging

Antibodies

Antibodies used

RBFOX2, Sigma, #006240, Prestige Antibodies® Immunoblotting: 0.4 µg/mL
SRSF1, mAb AK96 culture supernatant (Cáceres et al. 1997), Immunoblotting: 1:1000
SRSF6, mAb 8-1-28 culture supernatant (Fu and Maniatis 1990), Immunoblotting: 1:1000
Tubulin, Abcam, #ab6160, [YL1/2], Immunoblotting: 1:10000
Flag, Sigma, # F3165, clone M2, Immunoblotting: 10 µg/mL
GAPDH, Sigma, # G9545, polyclonal, Immunoblotting: 0.2 µg/mL
β-catenin, Abcam, #ab6302, polyclonal, Immunoblotting: 1:4000
β-Actin, Santa Cruz, #sc-1616, I-19, Immunoblotting: 1:1000
total-MEK 1/2, Cell Signaling, #8727, D1A5, Immunoblotting: 1:1000
Paxillin, BD Biosciences, BD612405, Clone 349/Paxillin (RUO), Immunofluorescence: 1:1000
Rac1, Cytoskeleton, Inc., ARC03, Immunoblotting: 1:500
A-Raf Santa Cruz, sc-408, C-20, Immunoblotting: 1:500
GFP abcam #ab6673 Immunohistochemistry 1:1000
Anti-FLAG Sigma #A2220 M2 Affinity gel Immunoprecipitation 30 µg/µL
ImmPRESS HRP anti-Goat IgG polymer) Vector Laboratories #MP-7401
Peroxidase-conjugated AffiniPure Goat Anti-Mouse IgG (H+L), Jackson ImmunoResearch Inc.,# 115-035-003, Immunoblotting: 1:10000
Peroxidase-conjugated AffiniPure Goat Anti-Rabbit IgG (H+L), Jackson ImmunoResearch Inc.,# 111-035-003, Immunoblotting: 1:10000
Peroxidase-conjugated AffiniPure donkey Anti- goat IgG (H+L), Jackson ImmunoResearch Inc., # 705-035-003, Immunoblotting: 1:10000
Alexa Fluor® 488 AffiniPure Goat Anti-Mouse IgG (H+L), Jackson ImmunoResearch Inc., 115-545-003, Immunofluorescence: 1:800
Donkey Anti-Rat IgG H&L (HRP) preadsorbed, Abcam, ab102265, Immunoblotting: 1:10000

Validation

All antibodies were validated by the supplier on human samples. All antibodies were checked in the lab by immunoblotting using cell lysates and compared to the supplier's.

Eukaryotic cell lines

Policy information about cell lines

Cell line source(s)

X50, X139, X252, were provided by Dr. Talia Golan (PMID: 28489577) (PMID: 29396858). BxPC3 (CRL-1687), HEK293T (CRL-3216), Phoenix-AMPHO (CRL-3213) and HEK293 (CRL-1573) cells lines were originally obtained from the American Type Culture Collection (ATCC).

Authentication

Cell line authentication test was performed at the Technion Genomics Center. The test was performed using the Promega GenePrint 24 System in order to determine short tandem repeat (STR) profile of 23 loci plus Amelogenin for gender determination (X or XY). In addition, the male-specific DYS391 locus is included to identify null Y allele results for Amelogenin.

Mycoplasma contamination

All cell lines are frequently tested for mycoplasma contamination. Cell lines used in this study were verified to be mycoplasma negative before undertaking any experiments .

Commonly misidentified lines
(See ICLAC register)

None

Animals and other organisms

Policy information about studies involving animals; ARRIVE guidelines recommended for reporting animal research

Laboratory animals

NOD SCID mice (Jackson Lab, 0001303) were ordered at 6 weeks of age. The mice were housed under standard laboratory conditions in specific-pathogen-free cages in an animal room at constant temperature (19–23 °C) and regulated humidity under a 12 h/12 h light–dark cycle and received standard laboratory chow and water ad libitum. All mice entered the experiments at 8–12 weeks of age. Both male and female mice were used for the experiments.

Wild animals

The study did not involve wild animals.

Field-collected samples

The study did not involve samples collected from the field.

Ethics oversight

PDA patient-derived xenograft (PDX) generation in nude mice were performed in accordance with the guidelines of Sheba Medical Center Institutional Animal Care and Use Committee (IACUC) (5539/13).
Metastatic and tumor formation in vivo experiments were performed in accordance with the guidelines of IACUC at the Hebrew University (MD-15-14634-5).

Note that full information on the approval of the study protocol must also be provided in the manuscript.
