# Supplementary material for: RBFOX2 modulates a metastatic signature of alternative splicing in pancreatic cancer
Source: Nature. 2023 Mar 22;617(7959):147–53. doi: 10.1038/s41586-023-05820-3 (PMC10156590; doi:10.1038/s41586-023-05820-3)
Supplement: Supplementary file 1 [file 41586_2023_5820_MOESM1_ESM.pdf]

nature portfolio

https://doi.org/10.1038/s41586-023-05820-3

Supplementary information

RBFOX2 modulates a metastatic signature of alternative splicing in pancreatic cancer

In the format provided by the authors and unedited

Supplementary information

RBFOX2 modulates a metastatic signature of alternative splicing in pancreatic cancer

Amina Jbara[1], Kuan-Ting Lin[2], Chani Stossel[3], Zahava Siegfried[1], Haya Shqerat[1], Adi Amar-Schwartz[1], Ela Elyada[1], Maxim Mogilevsky[1], Maria Raitses-Gurevich[3], Jared L. Johnson[6,7], Tomer M. Yaron[6,7,8,9], Ofek Ovadia[1], Gun Ho Jang[4], Miri Danan-Gothold[5], Lewis C. Cantley[6,7], Erez Y. Levanon[5], Steven Gallinger[4], Adrian R Krainer[2], Talia Golan[3] and Rotem Karni[1]

[1] Department of Biochemistry and Molecular Biology, Institute for Medical Research Israel-Canada, Hebrew University-Hadassah Medical School, Jerusalem, 91120, Israel.

[2] Cold Spring Harbor Laboratory, Cold Spring Harbor, NY 11724, USA.

[3] Division of Oncology, Sheba Medical Center Tel Hashomer, Ramat-Gan 5262100, Israel

[4] Department of Surgery, University of Toronto, Toronto, ON M5T 1P5, Canada.

[5] The Mina & Everard Goodman Faculty of Life Sciences, Bar-Ilan University, Ramat-Gan, 52900, Israel.

[6] Meyer Cancer Center, Weill Cornell Medicine, New York, NY 10021, USA.

[7] Department of Medicine, Weill Cornell Medicine, New York, NY 10021, USA.

[8] Englander Institute for Precision Medicine, Institute for Computational Biomedicine, Weill Cornell Medicine, New York, NY 10021, USA.

[9] Department of Physiology and Biophysics, Weill Cornell Medicine, New York, NY 10065, USA.

Correspondence:
Rotem Karni
(e-mail: rotemka@ekmd.huji.ac.il)

Table of Contents

Supplementary figure 1. Raw data of immunoblot scans from Figure.1 3

Supplementary figure 2. Raw data of immunoblot scans from Figure.2 4

Supplementary figure 3. Raw data of PCR gels scans from Figure.4 5

Supplementary figure 4. Raw data of immunoblot scans from extended data figure.3 6

Supplementary figure 5. Raw data of immunoblot scans from extended data fig.4h and k ..7

Supplementary figure 6. Raw data of PCR gels scans from extended data figure.6..............8

Supplementary figure 7. Raw data of immunoblot scans from extended data figure.8 11

Supplementary figure 8. Raw data of PCR gels scans from extended data figure.9............12

Supplementary figure 9. Raw data of immunoblot scans from extended data figure.9 13

Supplementary figure 10. Raw data of PCR gels scans from extended data figure.11.........14

Supplementary figure 11. Raw data of PCR gels scans from extended data figure.12.........15

Supplementary figure 12. Lung histology samples related to figure 3g. 16

Supplementary figure 13. Lung histology samples related to figure 4h. 17

Supplementary figure 14. Lung histology samples related to figure 4l. 18

Supplementary figure 15. Lung histology samples related to extended data figure 8a. 19

Supplementary figure 16. Lung histology samples related to extended data figure 8p. 20

Supplementary figure 17. Lung histology samples related to extended data figure 11g. 21

Supplementary figure 18. Lung histology samples related to extended data figure 12g. 22

Supplementary Table 1. Genetic alterations and clinical data of PDA patient. 23

Supplementary Table 2. Differentially spliced events in PDA patient samples, primary tumors versus metastatic tumors. 23

Supplementary Table 3. Sequence motif enrichment analysis..23

Supplementary Table 4. Reactome analysis. 23

Supplementary Table 5. Differentially gene expression changes in PDA patient samples, primary tumors versus metastatic tumor. 23

Supplementary Table 6. Differentially spliced events in RBFOX2 manipulated cell lines..23

Supplementary Table 7. Comparisons of RBFOX2 target genes to known RBFOX2 target genes. 23

Supplementary Table 8. Serine-threonine kinome analysis for MPRIP isoforms. 23

Supplementary Table 9. Mass spectrometry analysis for MPRIP isoforms. 23

Supplementary Table 10. sgRNAs sequences 24

Supplementary Table 11. PCR primers sequences 25

RT-PCR primers for splicing validation..25

Supplementary Table 12. Primary and secondary antibodies 26

Supplementary figure 1. Raw data of immunoblot scans from Figure.1

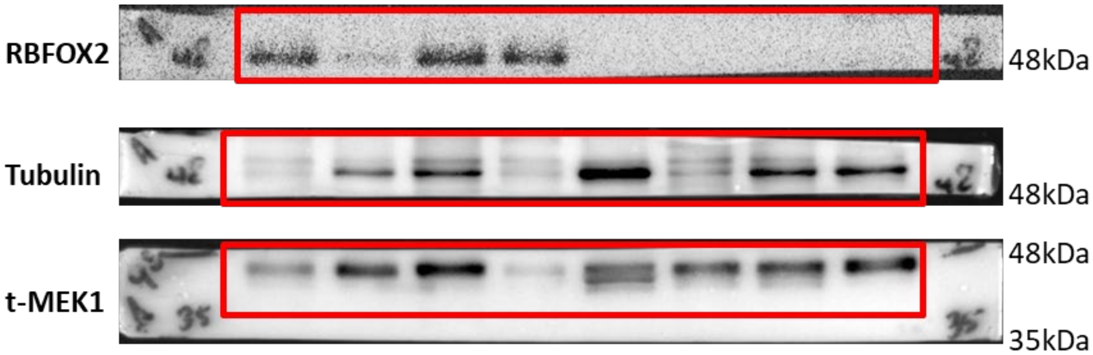

Fig. 1e

Supplementary figure 2. Raw data of immunoblot scans from Figure.2

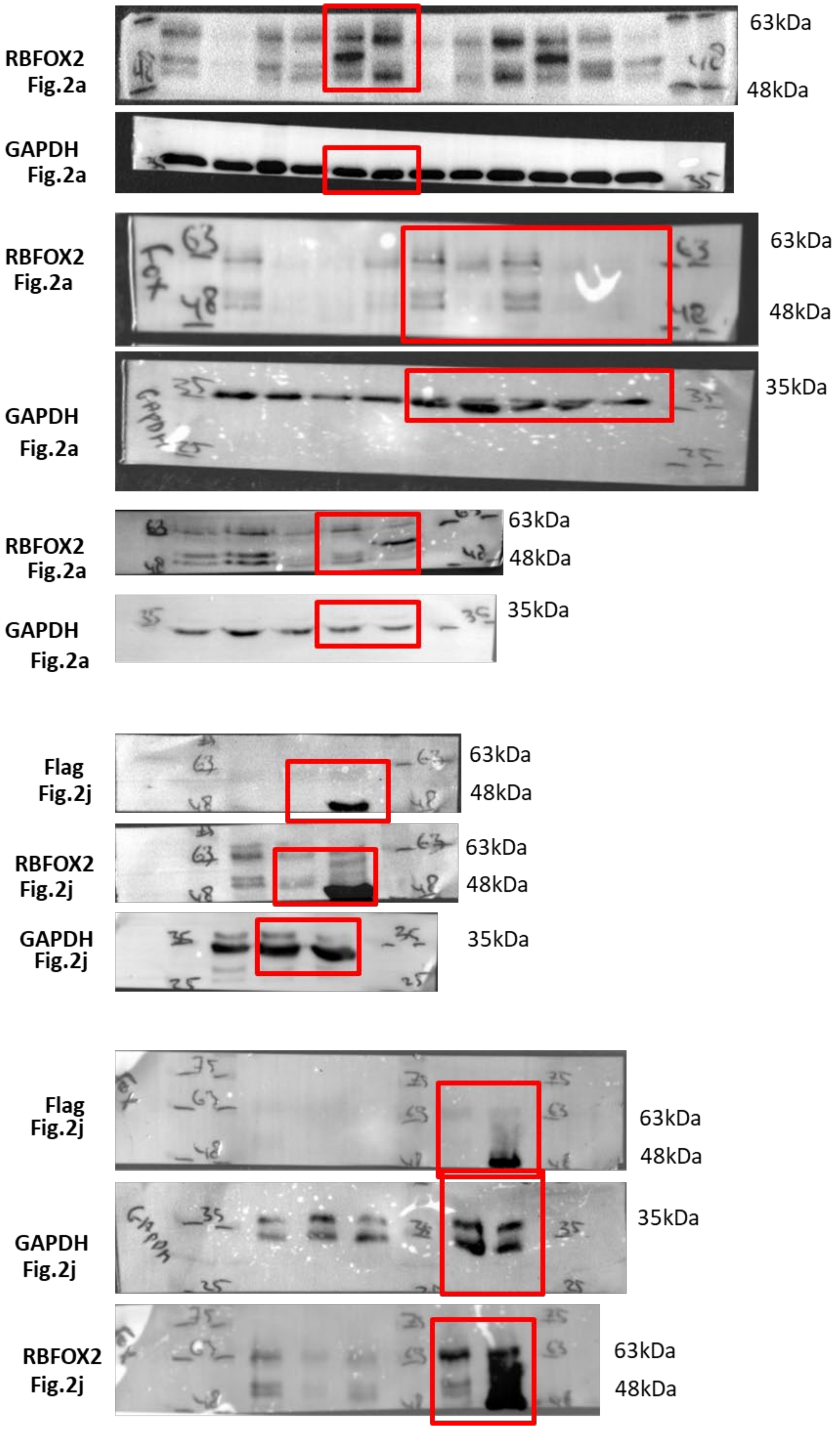

Supplementary figure 3. Raw data of PCR gels scans from Figure.4

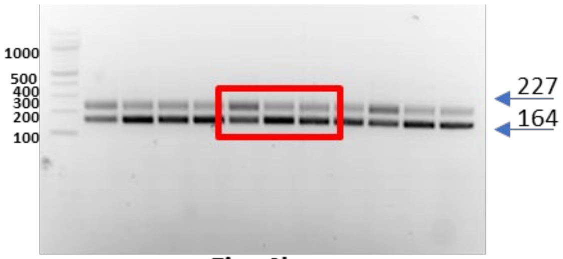

Fig. 4b

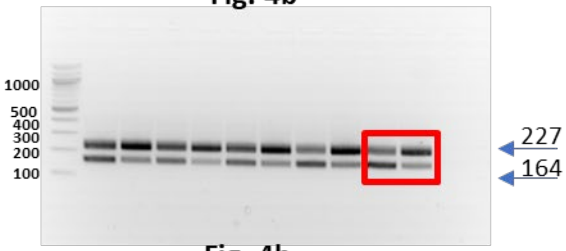

Fig. 4b

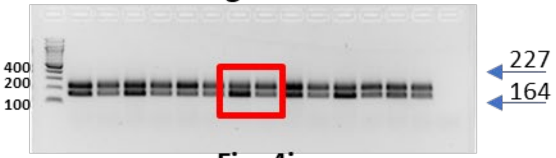

Fig. 4i

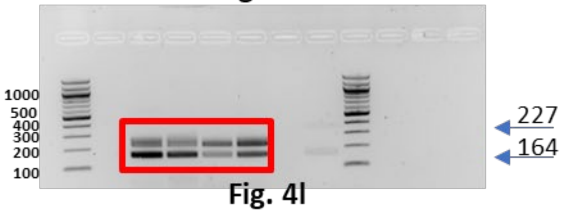

Fig. 4l

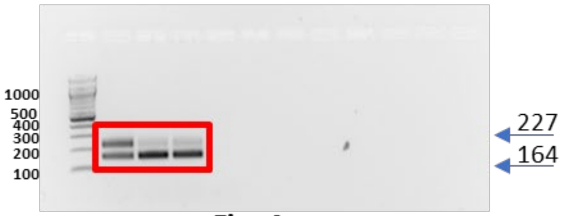

Fig. 4e

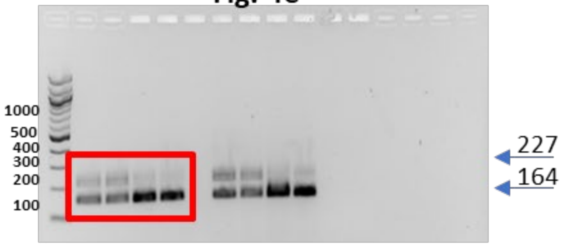

Fig. 4h

Supplementary figure 4. Raw data of immunoblot scans from extended data figure.3

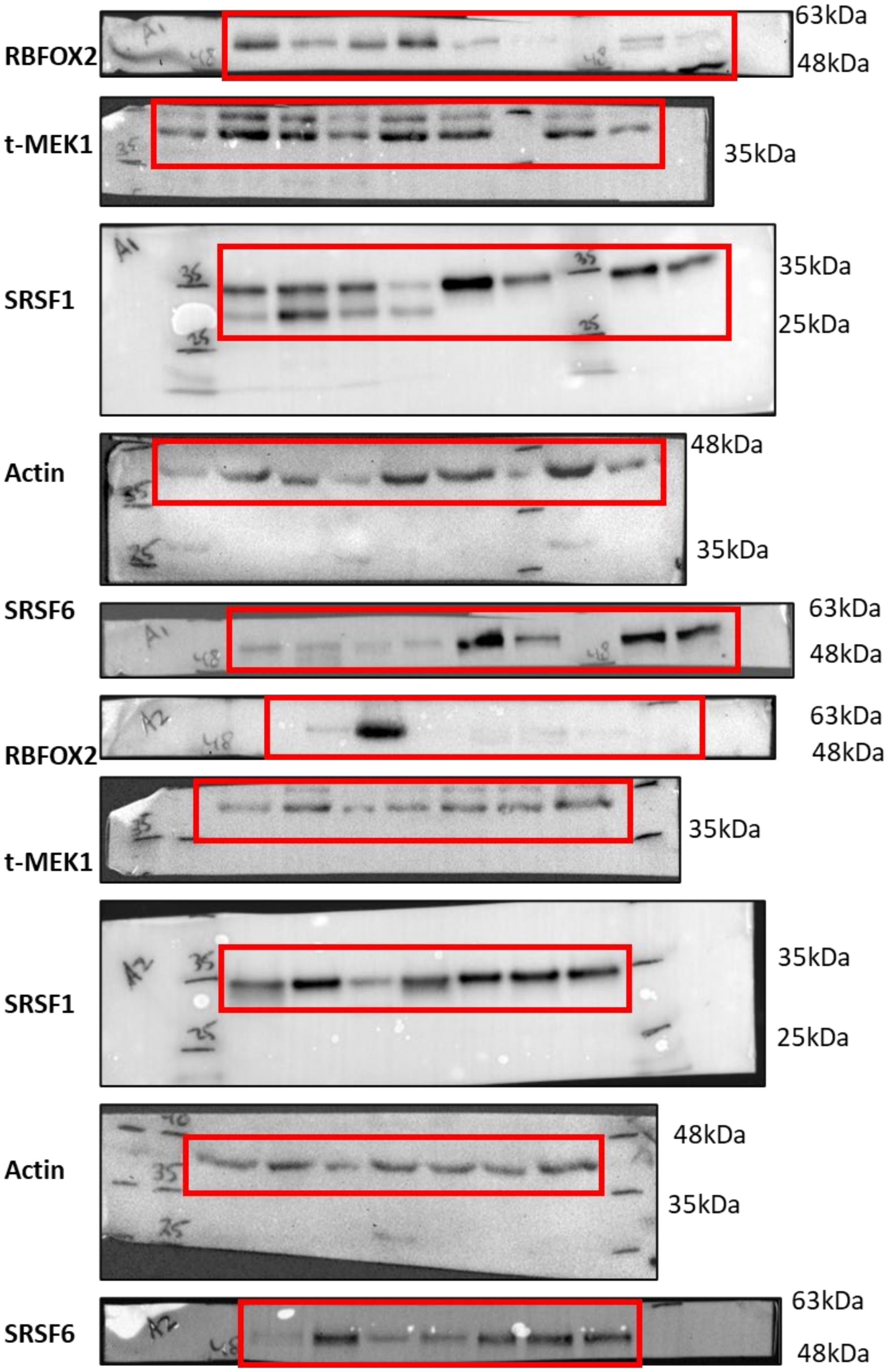

Extended Data Figure.3a

Supplementary figure 5. Raw data of immunoblot scans from extended data fig.4

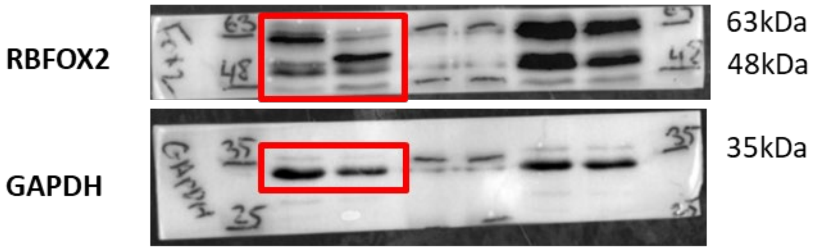

Extended Data Fig.4a

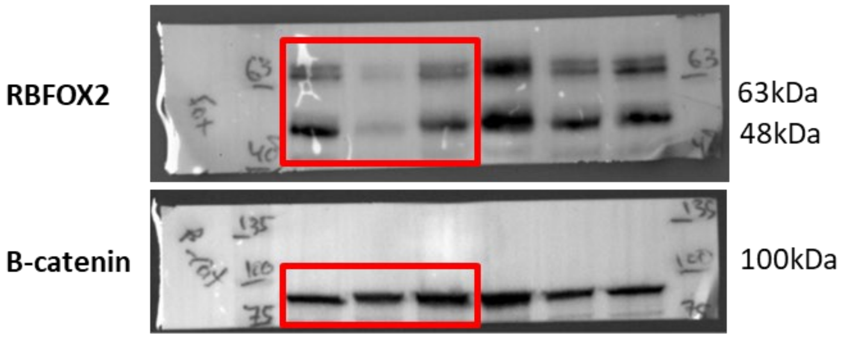

Extended Data Fig.4f

Supplementary figure 6. Raw data of PCR gels scans from extended data figure.6

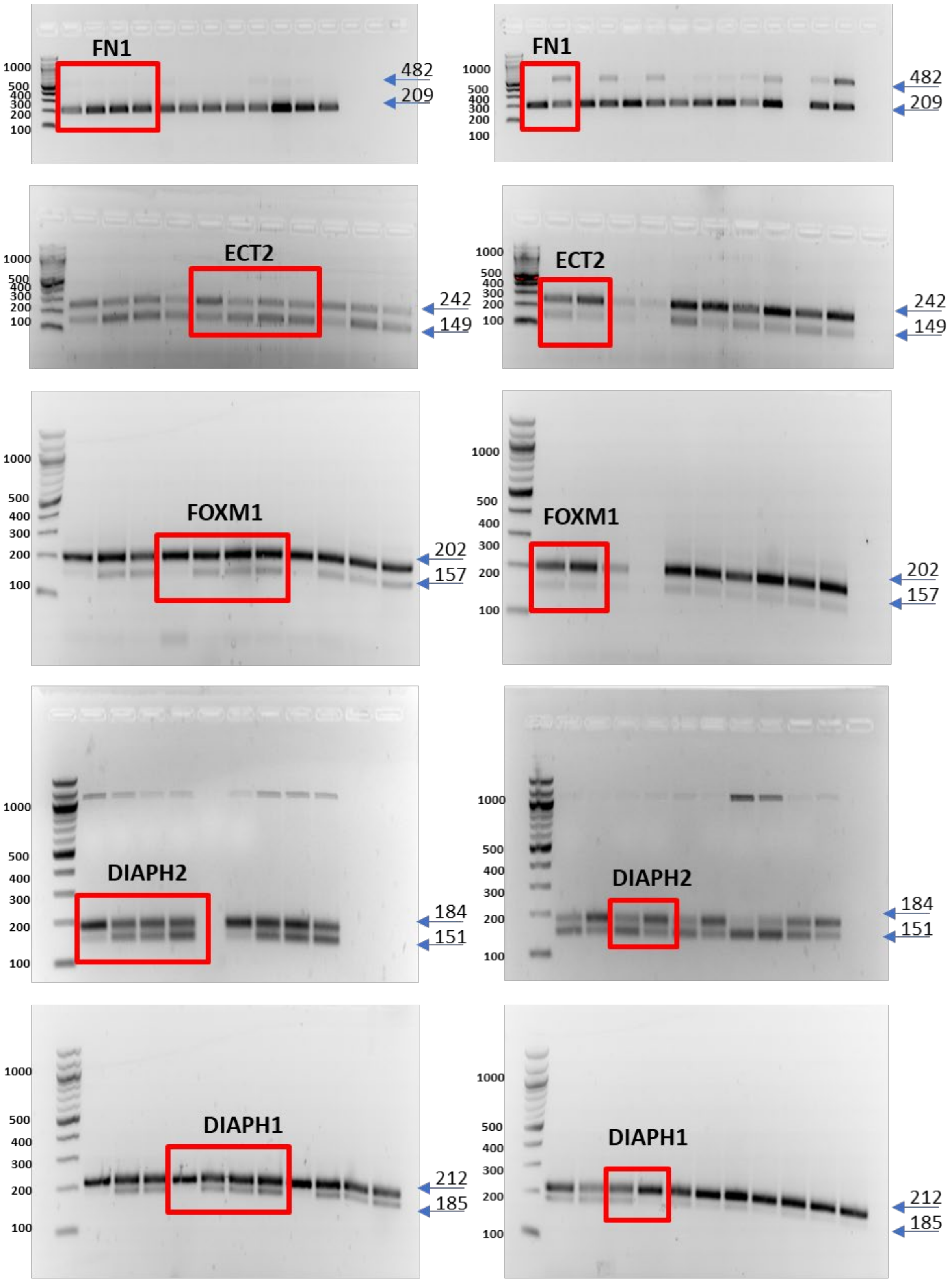

Supplementary figure 6. Raw data of PCR gels scans from extended data figure.6

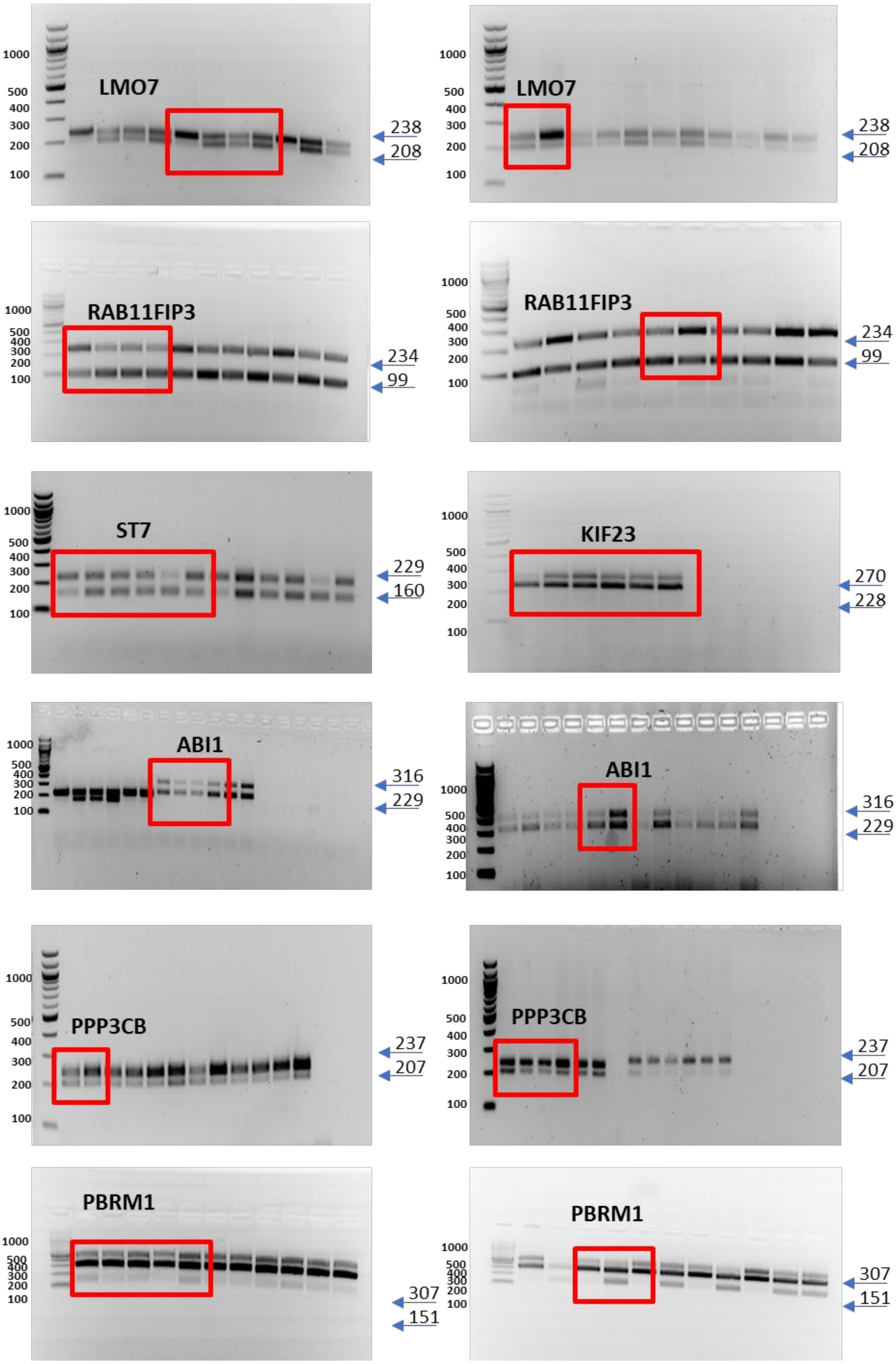

Supplementary figure 6. Raw data of PCR gels scans from extended data figure.6

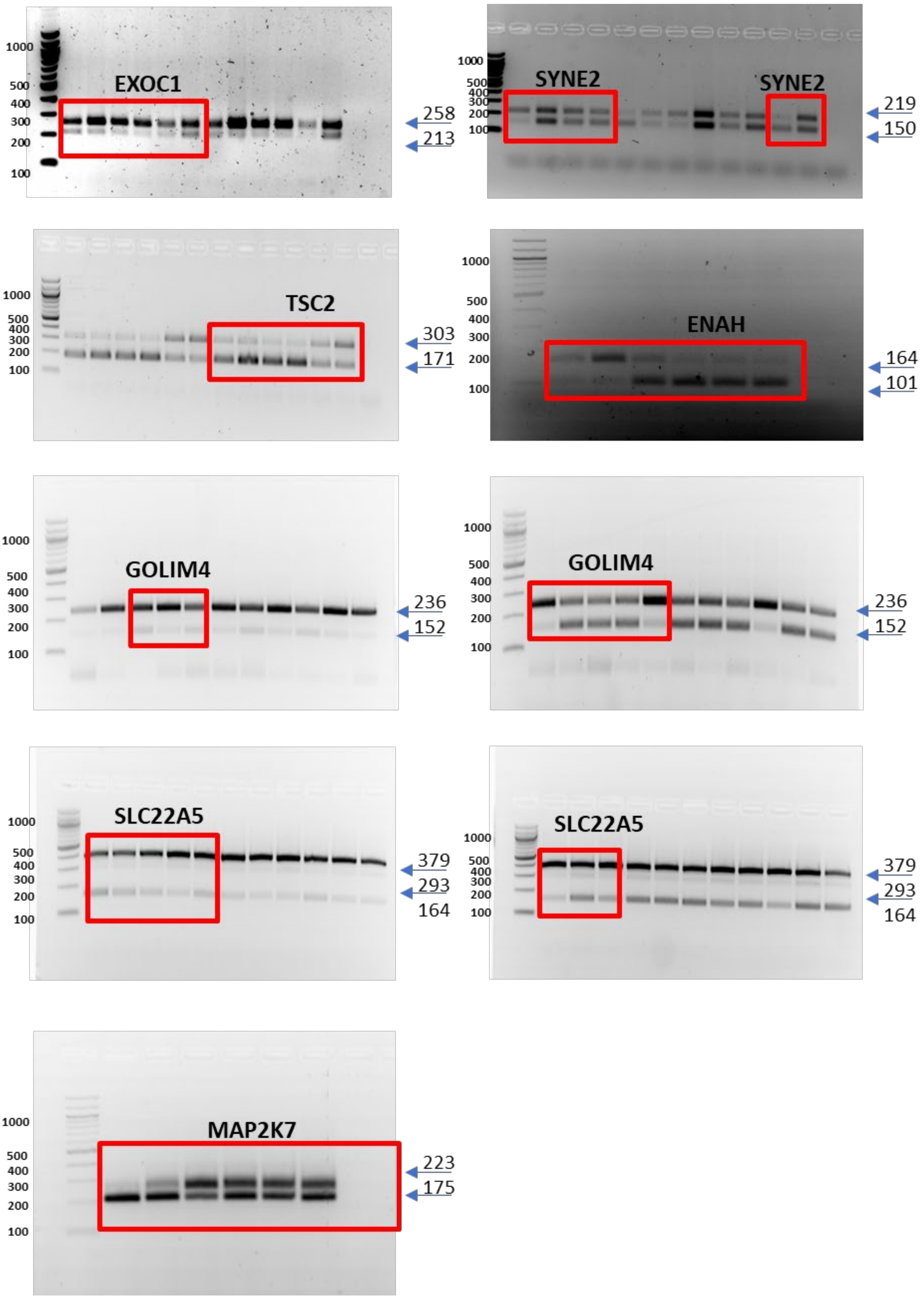

Supplementary figure 7. Raw data of immunoblot scans from extended data figure.8

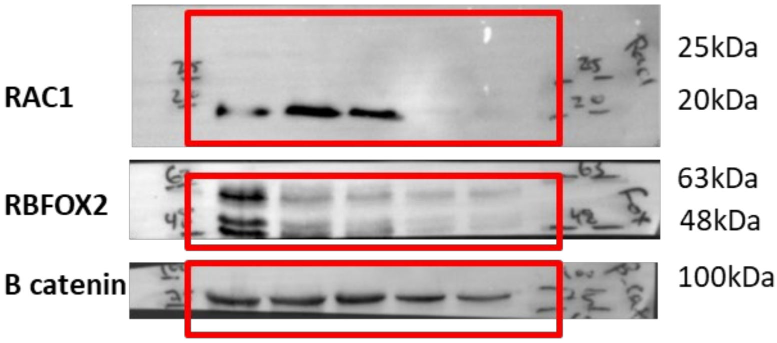

Extended Data Fig.8m

Supplementary figure 8. Raw data of PCR gels scans from extended data figure.9

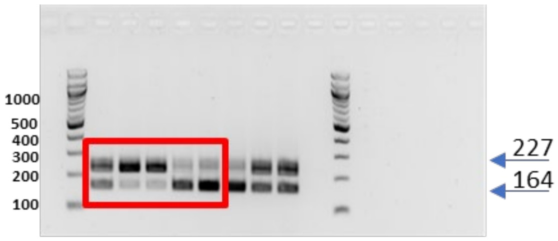

Extended Data Fig.9e

Supplementary figure 9. Raw data of immunoblot scans from extended data figure.10

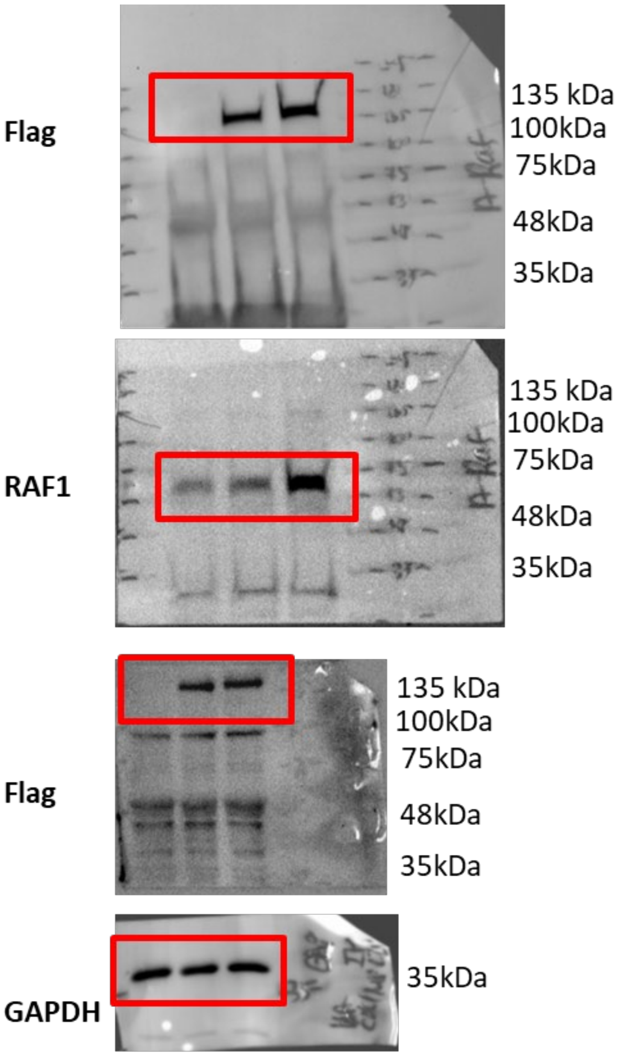

Extended Data Fig.10h

Supplementary figure 10. Raw data of PCR gels scans from extended data figure.11

Extended Data Fig.11a

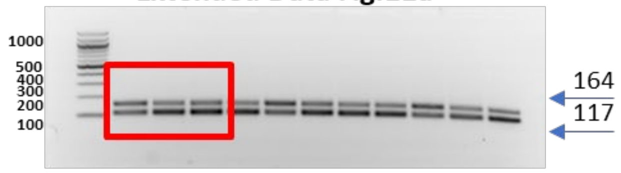

Extended Data Fig.11a

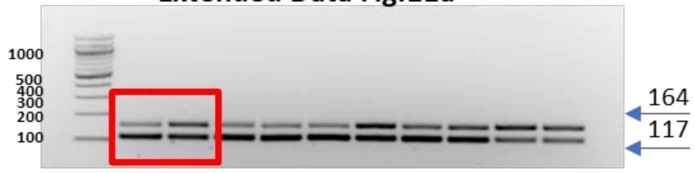

Extended Data Fig.11c

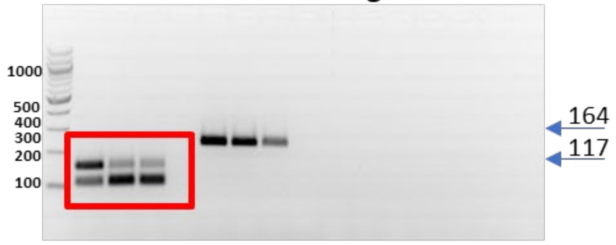

Extended Data Fig.11e

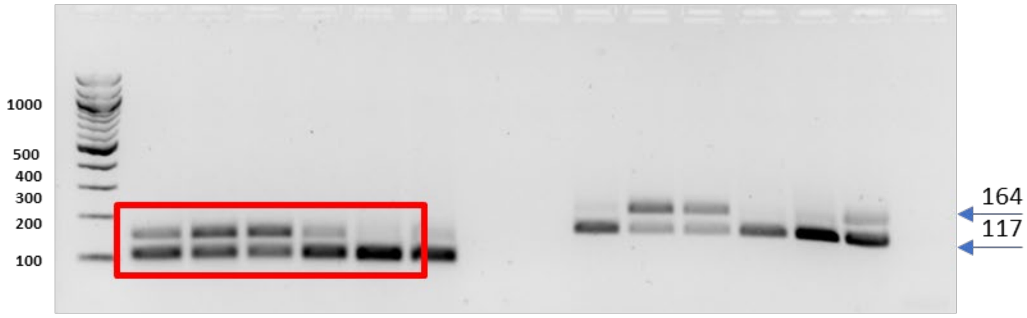

Extended Data Fig.11g

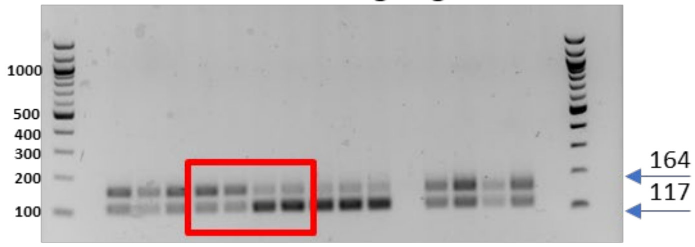

Supplementary figure 11. Raw data of PCR gels scans from extended data figure.12

Extended Data Fig.12a

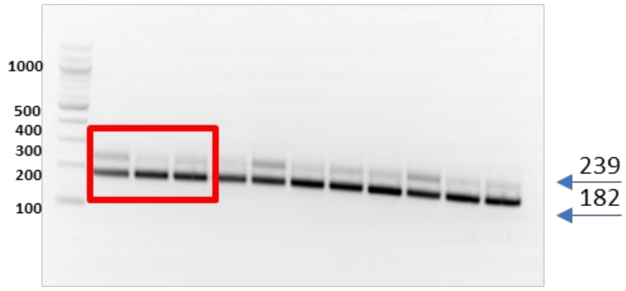

Extended Data Fig.12a

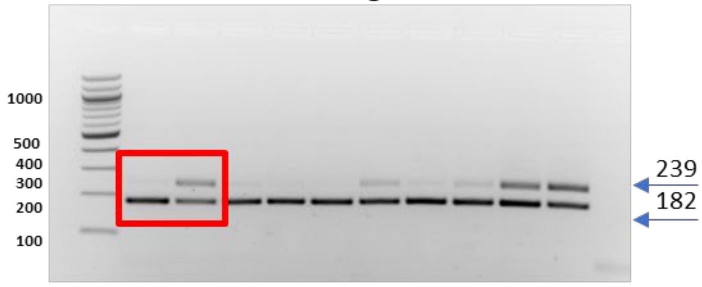

Extended Data Fig.12c

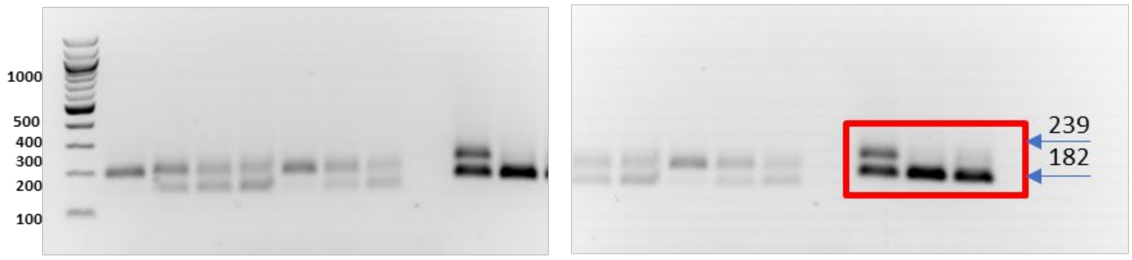

Extended Data Fig.12e

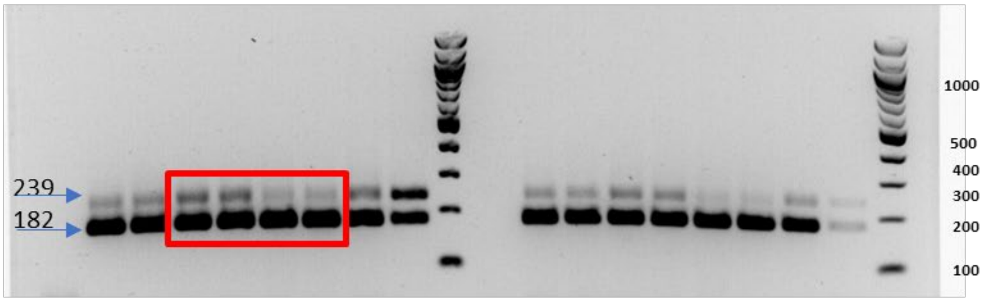

Extended Data Fig.12g

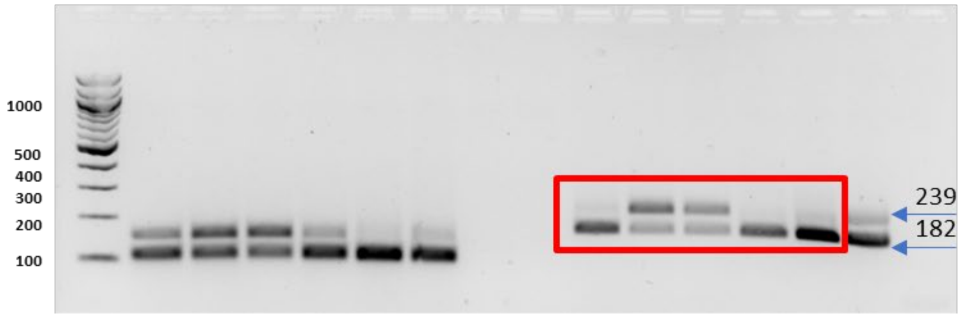

Supplementary figure 12. Lung histology samples related to figure 3g.
(n=7 mice/group)

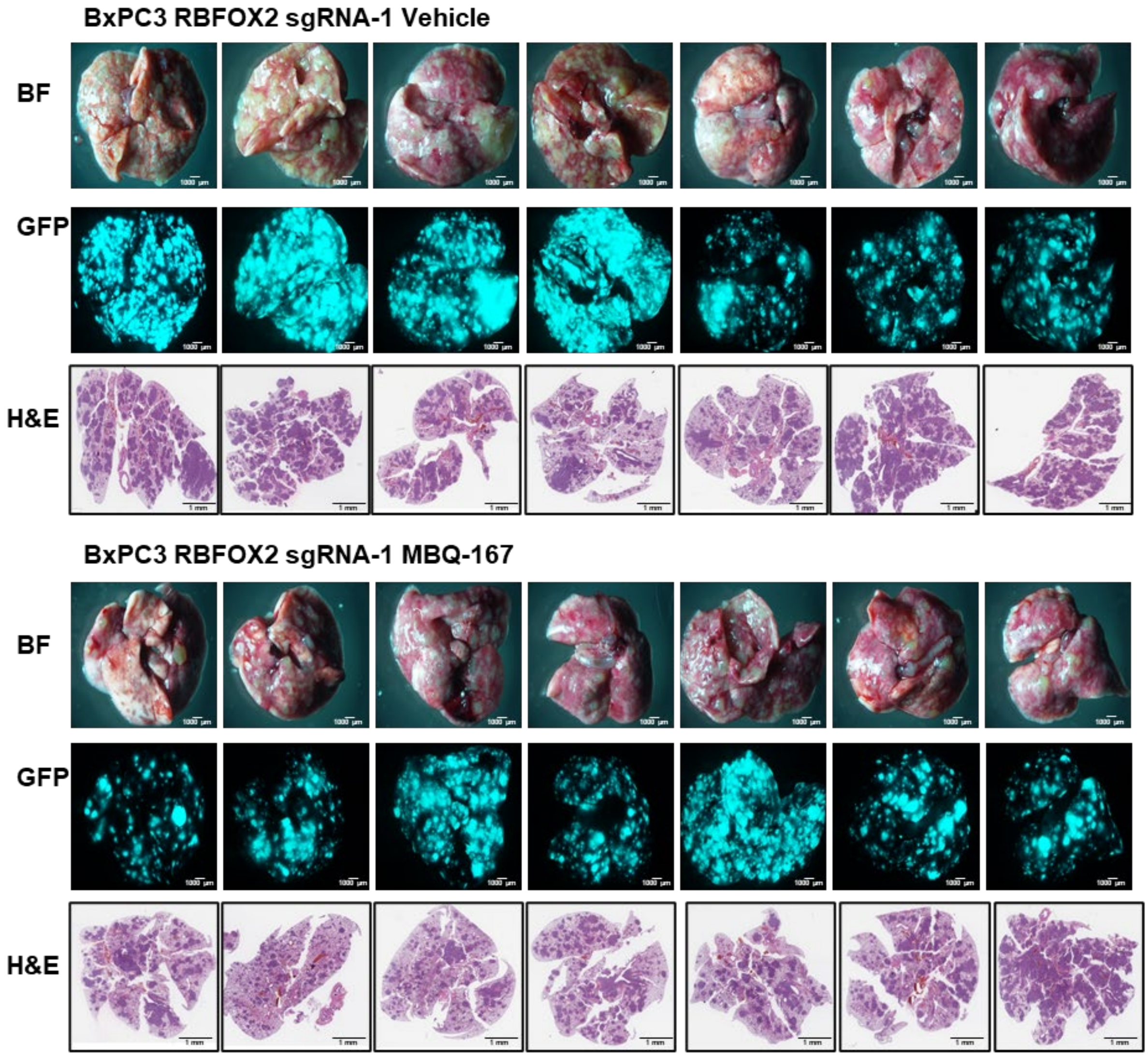

Supplementary figure 13. Lung histology samples related to figure 4h. (n=4 mice for CRISPR Cont. and, n=5 mice for 5'ss MPRIP sgRNA).

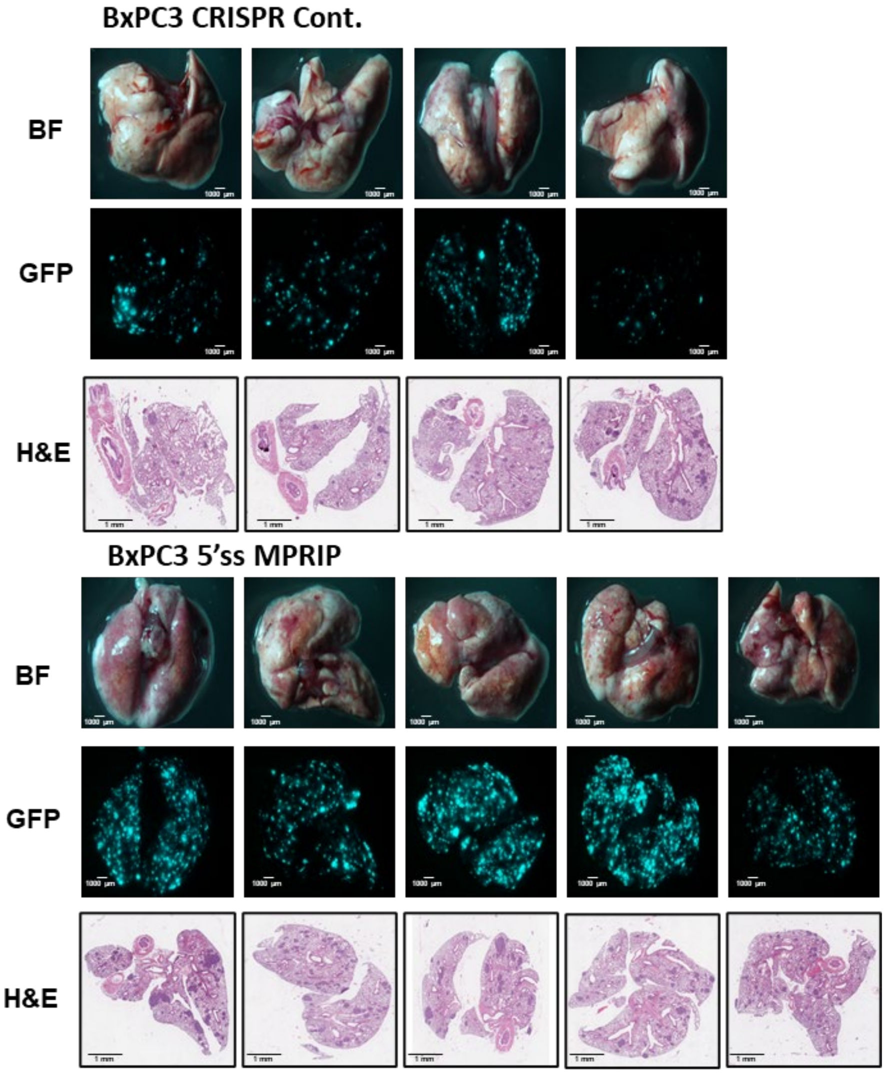

Supplementary figure 14. Lung histology samples related to figure 4l (n=10 mice/ group)

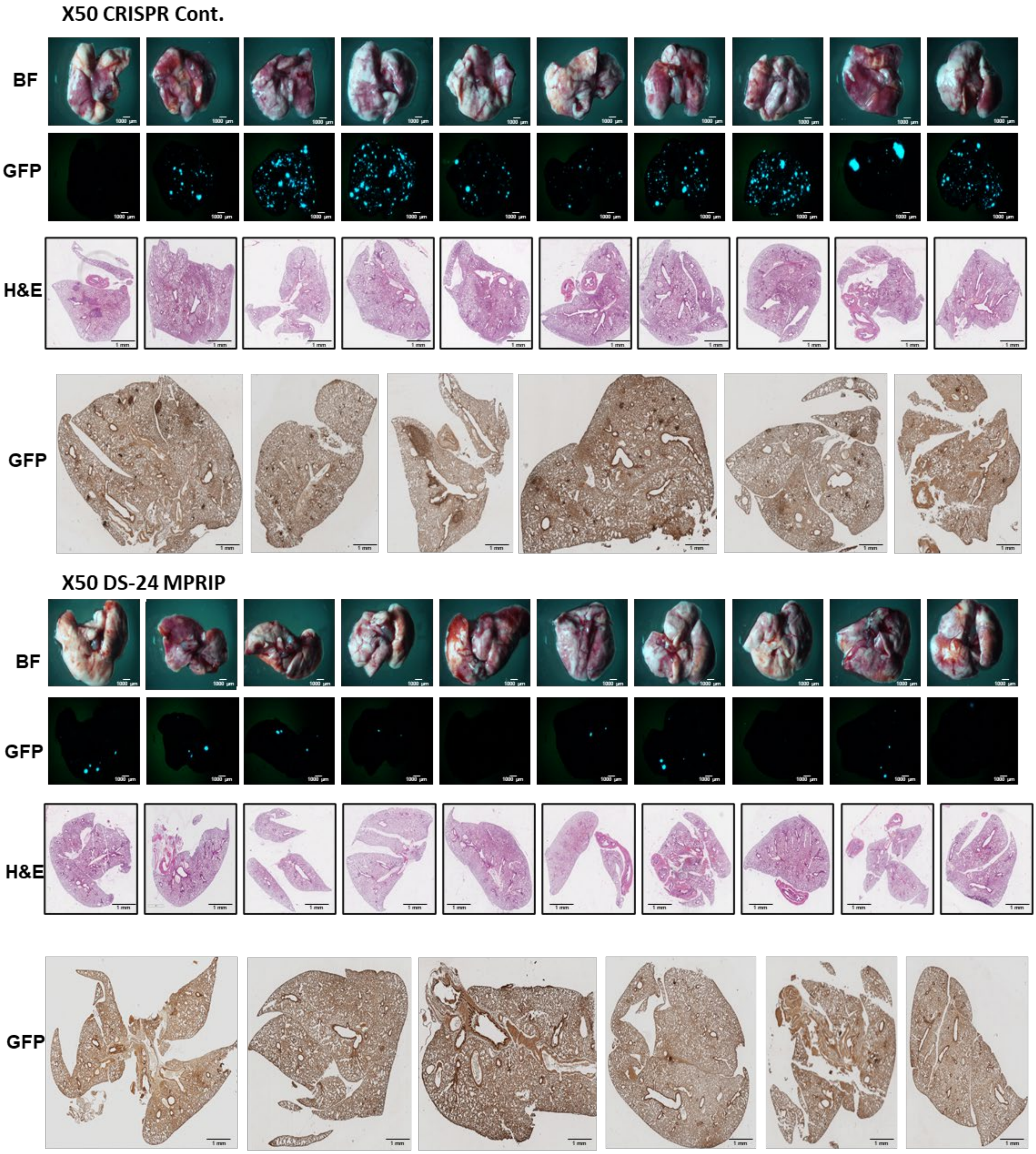

Supplementary figure 15. Lung histology samples related to extended data figure 8a. (n=8 mice/group)

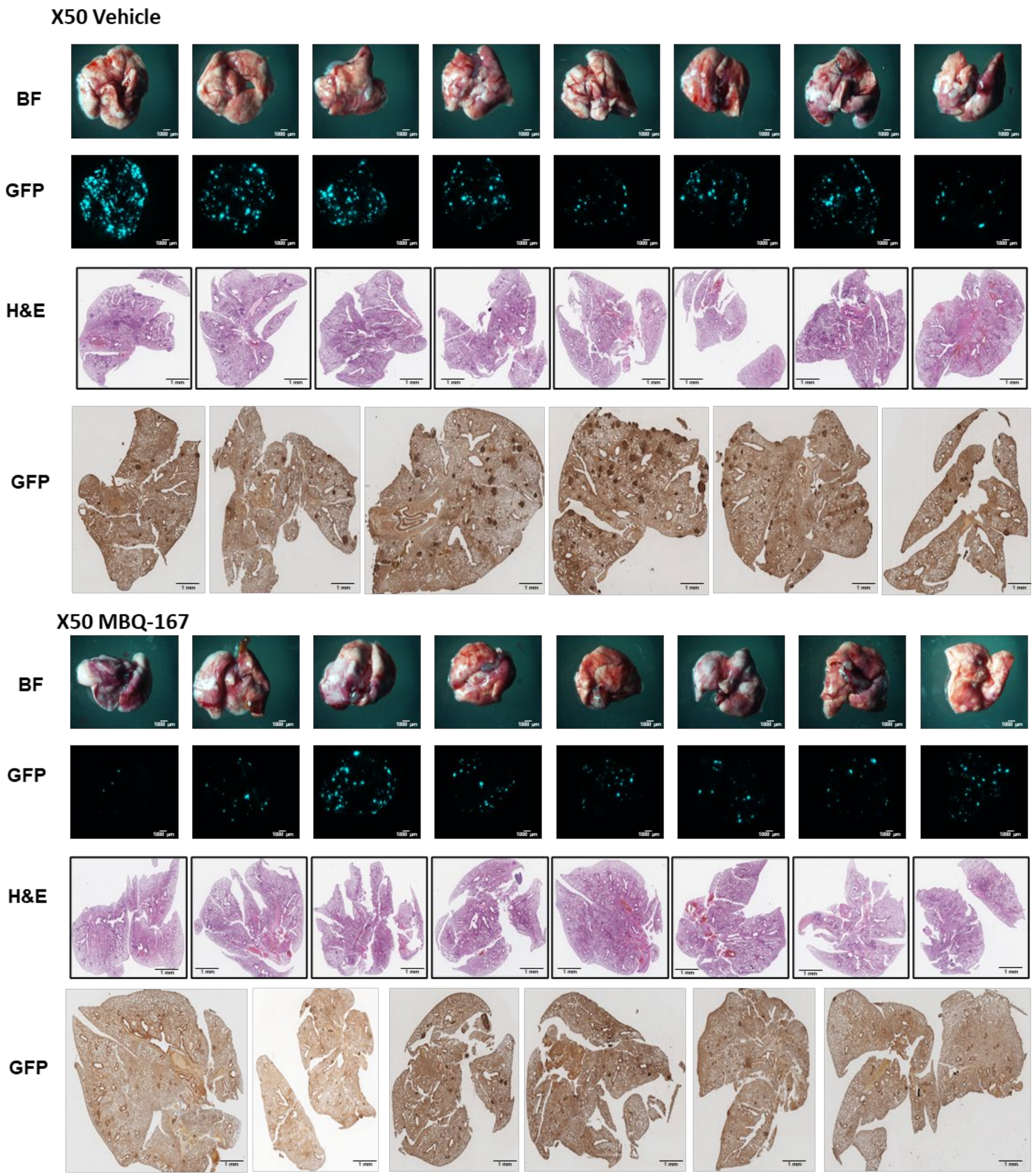

Supplementary figure 16. Lung histology samples related to extended data figure 8p (n=9 for CRISPR Cont, n=7 sgRNA-1 Rac1)

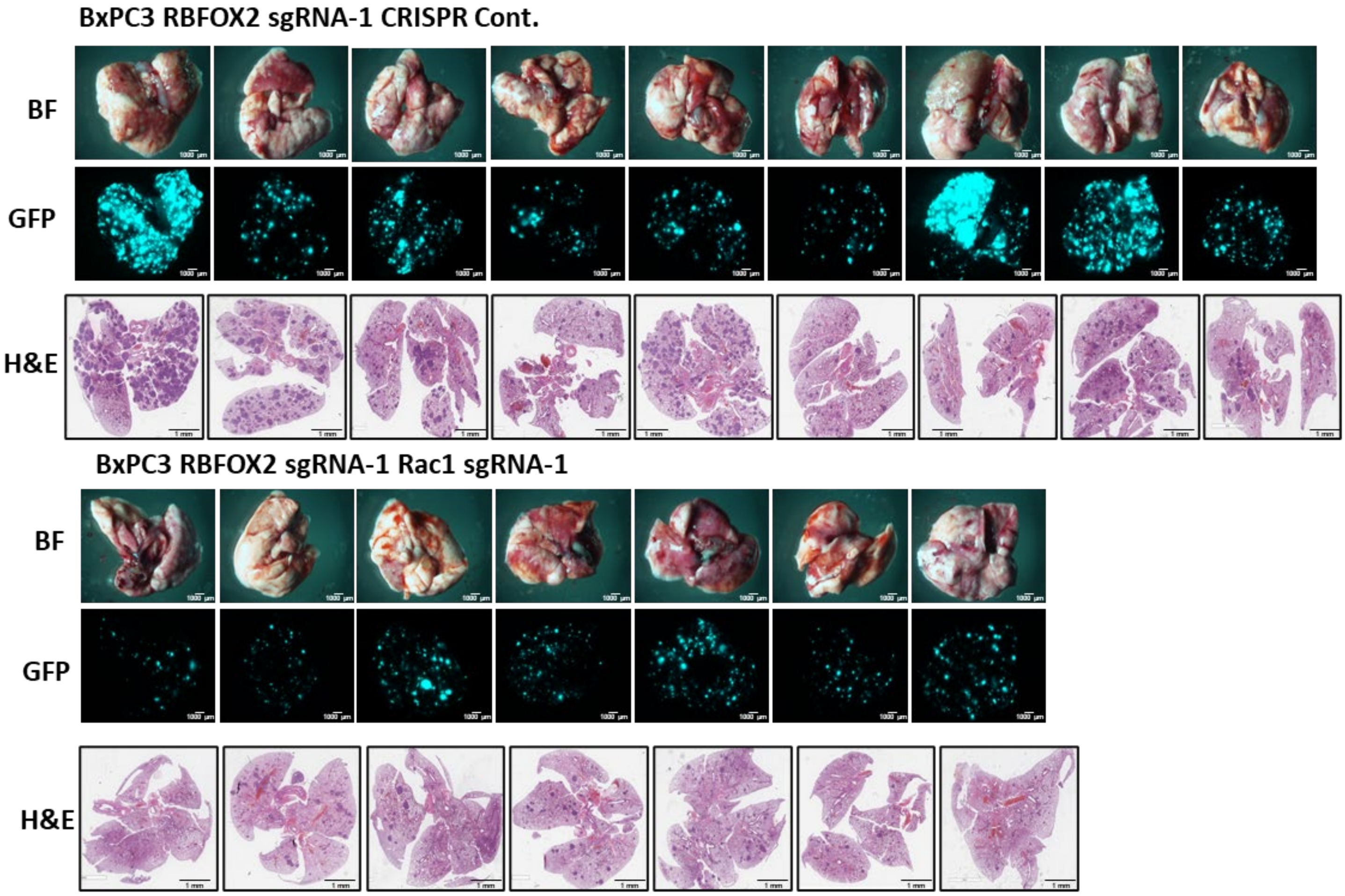

Supplementary figure 17. Lung histology samples related to extended data figure 11g. (n=8 mice/group for CRISPR Cont. and 3' ss MYL6 sgRNAs)

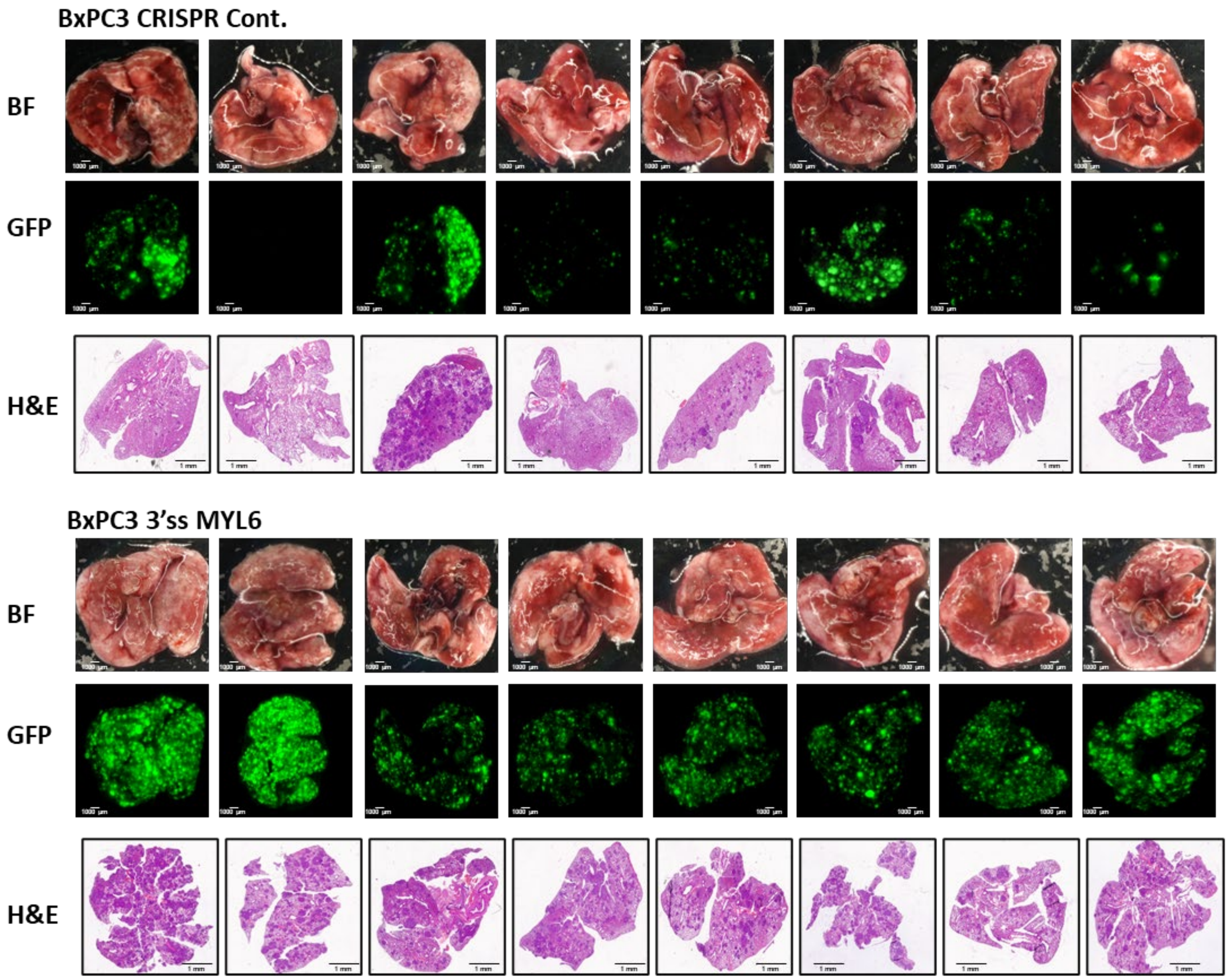

Supplementary figure 18. Lung histology samples related to extended data figure 12g. (n=9 mice/group for CRISPR Cont. and 3' ss CLSTN1 sgRNAs)

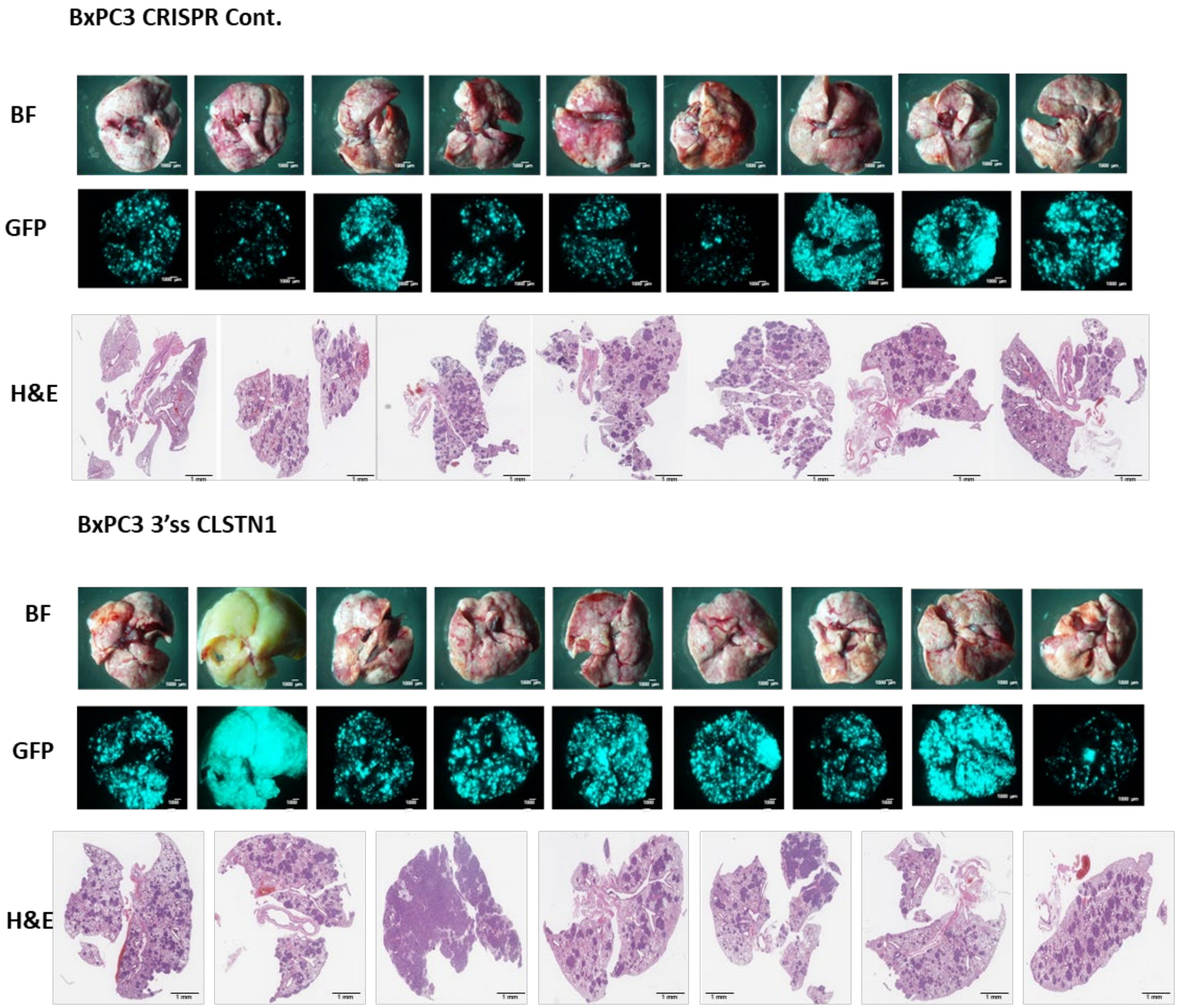

Supplementary Table 1. Genetic alterations and clinical data of PDA patients.
Related to figs.1a-b.

Supplementary Table 2. Differentially spliced events in PDA patient samples, primary tumors versus metastatic tumors.
Related to fig.1b .

Supplementary Table 3. Sequence motif enrichment analysis.
Related to fig.1d and extended data figs.2a and 5f.

Supplementary Table 4. Reactome analysis.
Related to figs.1c,3c and extended data figs.2b and 5d-e.

Supplementary Table 5. Differentially gene expression changes in PDA patient samples, primary tumors versus metastatic tumor.
Related to extended data fig.1h.

Supplementary Table 6. Differentially spliced events in RBFOX2 manipulated cell lines.
manipulated cell lines: (X50 Pwzl(-) vs. X50 OE RBFOX2) vs. (BxPC3 CRISPR Cont. vs. BxPC3 sgRNA-1/2 RBFOX2). Related to figs. 3a-b and extended data figs.5a-c.

Supplementary Table 7. Comparisons of RBFOX2 target genes to known RBFOX2 target genes.
Related to extended data figs.1b and 5d-e.

Supplementary Table 8. Serine-threonine kinome analysis for MPRIP isoforms.
Related to extended data fig.9b.

Supplementary Table 9. Mass spectrometry analysis for MPRIP isoforms.
Related to extended data figs.9c-g.

Supplementary Table 10. sgRNAs sequences

**RBFOX2 sgRNA-1**	GTGGGAATTCCATTCTGCGG
**RBFOX2 sgRNA-2**	GAAGGTGGAGCACAGACAGA
**sgRNA control**	GGTGTGCGTATGAAGCAGTG
**Target sequence for RBFOX2 EIJ sgRNA**	TTTGCTTAGCAGACAGAAGG
**MPRIP exon 23 3'ss sgRNA**	GCTCAATTACGGACTGTCAG
**MPRIP exon 23 5'ss sgRNA**	CGCTTCCCGGCCCATGCAGG
**MYL6 exon 6 3'ss sgRNA**	ATATGCCTCACAAACGCTGC
**CLSTN1 exon 10 5'ss sgRNA**	CTGTGGCCTCTGCAGGTTGC
**CLSTN1exon 10 3'ss sgRNA**	TTCAACTCCTGAAAACTCTG
**DS-24 MPRIP sgRNA**	CTGTGGGCATGGAATGCGTG
**Rac1 sgRNA-1**	ACACTTGATGGCCTGCATCA
**Rac1 sgRNA-2**	ATTTAAGATACTTACACAGT

Supplementary Table 11. PCR primers sequences

RT-PCR primers for splicing validation	
**MPRIP**	AAAGCAACCCTGACTTCTTGA
	CTCAACTTGGATGGGACACA
**MYL6**	GCATGAGGACAGCAATGGTT
	CAAAATTCACACAGGGAAAGG
**CLSTN1**	AATGGCACCACTACGTCCTC
	TCGGAAAAACTGGGTCATGT
**FOXM1**	GACATGTTTGTCCGGGAGAC
	GGAGTTCGGTTTTGATGGTC
**LMO7**	ACTGCGTTACCCTTCAATCG
	GCTGACGCAAGTTTTGAACA
**RAB11FIP3**	GCTGTCCCCAGAGACCCTAT
	GCCACCTTCTTGCTGGAGAG
**GOLIM4**	GACAACTAAGGAAAGCACACCA
	CACCTGGGTCACATTTTGCT
**ECT2**	GAGATGCCTCAGATTGAAACAA
	TCAAATTCCGGAGAATCCAA
**MAP2K7**	AGGATCGACCTCAACCTGGA
	GGGGTGTGAACAGGGTTG
**ENAH**	CAAATACAATGAATGGCAGCA
	GTCAAGTCCTTCCGTCTGGA
**DIAPH1**	GACCCGGGACAAGAAGAAG
	ATGACTGTGCTGTGGGATCA
**KIF23**	TGCAAAGCAGAAGAGGTTGA
	GAAAACTTCAAAAGCCTCCTCA
**PBRM1**	GTGATTAAGGCCCAACACCC
	CTGGTGCTGGAGTCCCTAC
**SLC22A5**	GCAGTCCCACAACATTCTGG
	CTCGGCTGTGTACACGTAGA
**FN1**	TTGATAACCTGAGTCCCGGC
	TGAGTAACGCACCAGGAAGT
**ABI1**	TTCCCAGTATGGCACAATGA
	GGGAGGTGGAGAGTCATCAA
**ST7**	ATGCAGAAAGCCTGGAGAGA
	CCTTCAGGGCCTGCTTAAAT
**SYNE2**	CCTCTCACGAAGAGGACGAG
	CAGGTGGAACATTCCTGTCA
**TSC2**	CTCAACGAGAGACCCAAGAG
	AGCCGTGAAGTTGGAGAAGA
**PPP3CB**	ACAGGGATGTTGCCTAGTGG
	GTGGTTCTCAGTGGCATGTG
**EXOC1**	TGAAGTTGCAAAGATCAAGATGA
	TCAAATTTGGTCCTGTCAGC
**DIAPH2**	GCCAATGAAGAGGAAACGAA
	ATAATGGCTGAGCTGCAGGA

Quantitative RT-PCR primers	
**RBFOX2**	TGGAAATTAAGCCCAGTAGTTG
	TGATACCCCCTCTTCCTGA
**18S rRNA**	GTAACCCGTTGAACCCCATT
	CCATCCAATCGGTAGTAGCG

Supplementary Table 12. Primary and secondary antibodies

**RBFOX2**	Sigma	#006240	Prestige Antibodies® Immunoblotting	Immunoblotting: 0.4 µg/mL
**SRSF1**		(Cáceres et al. 1997)	mAb AK96 culture supernatant	Immunoblotting: 1:1000
**SRSF6**		(Fu and Maniatis 1990)	mAb 8-1-28 culture supernatant	Immunoblotting: 1:1000
**Tubulin**	Abcam	#ab6160	[YL1/2]	Immunoblotting: 1:10000
**Flag**	Sigma	# F3165	clone M2	Immunoblotting: 10 µg/mL
**GAPDH**	Sigma	# G9545	polyclonal	Immunoblotting: 0.2 µg/mL
**β-catenin**	Abcam	#ab6302	polyclonal	Immunoblotting: 1:4000
**β-Actin**	Santa Cruz	#sc-1616	I-19	Immunoblotting: 1:1000
**total-MEK 1/2**	Cell Signaling	#8727	D1A5	Immunoblotting: 1:1000
**Paxillin**	BD Biosciences	BD6124 0	5, Clone 349/Paxillin (RUO)	Immunofluoresce nce: 1:1000
**Rac1**	Cytoskeleton, Inc.	ARC03		Immunoblotting: 1:500
**A-Raf**	Santa Cruz	sc-408	C-20	Immunoblotting: 1:500
**GFP**	abcam	#ab6673		Immunohistoche mistry 1:1000
**Anti-FLAG**	Sigma	#A2220	M2 Affinity gel	Immunoprecipita tion 30 µg/µL
**ImmPRESS HRP anti-Goat IgG polymer)**	Vector Laboratories	#MP-7401		
**Peroxidase-conjugated AffiniPure Goat Anti-Mouse IgG (H+L)**	Jackson ImmunoRese arch Inc.	# 115-035-003		Immunoblotting: 1:10000

**Peroxidase-conjugated AffiniPure Goat Anti-Rabbit IgG (H+L)**	Jackson ImmunoResearch Inc.	,# 111-035-003		Immunoblotting: 1:10000
**Peroxidase-conjugated AffiniPure donkey Anti- goat IgG (H+L),**	Jackson ImmunoResearch Inc.	# 705-035-003		Immunoblotting: 1:10000
**Alexa Fluor® 488 AffiniPure Goat Anti-Mouse IgG (H+L)**	Jackson ImmunoResearch Inc.	115-545-003		Immunofluorescence: 1:800
**Donkey Anti-Rat IgG H&L (HRP) preadsorbed**	Abcam	ab102265		Immunoblotting: 1:10000
